# Should Fertility Preservation Be Offered to Young Women with Melanoma Receiving Immune Checkpoint Inhibitors? A SWOT Analysis

**DOI:** 10.3390/curroncol32120702

**Published:** 2025-12-12

**Authors:** Diego Raimondo, Michele Miscia, Antonio Raffone, Manuela Maletta, Linda Cipriani, Paola Valeria Marchese, Francesca Comito, Rossella Vicenti, Federica Cortese, Enrico Pazzaglia, Linda Bertoldo, Luigi Cobellis, Renato Seracchioli

**Affiliations:** 1 Division of Gynaecology and Human Reproduction Physiopathology, IRCCS Azienda Ospedaliera-Universitaria di Bologna, 40138 Bologna, Italy; diego.raimondo2@unibo.it (D.R.); manuela.maletta2@unibo.it (M.M.); linda.cipriani@aosp.bo.it (L.C.); rossella.vicenti@unibo.it (R.V.); federica.cortese3@studio.unibo.it (F.C.); enrico.pazzaglia@studio.unibo.it (E.P.); linda.bertoldo@studio.unibo.it (L.B.); renato.seracchioli@unibo.it (R.S.); 2 Department of Medical and Surgical Sciences (DIMEC), University of Bologna, 40126 Bologna, Italy; paola.marchese8@unibo.it; 3 Department of Woman, Child and General and Specialized Surgery, University of Campania “Luigi Vanvitelli”, 80138 Naples, Italy; luigi.cobellis@unicampania.it; 4 Division of Medical Oncology, IRCCS Azienda Ospedaliera-Universitaria di Bologna, 40138 Bologna, Italy; francesca.comito@aosp.bo.it

**Keywords:** adolescents and young adults (AYA), melanoma, immunotherapy, immune checkpoint inhibitors, fertility preservation, controlled ovarian stimulation (COS), ovarian tissue cryopreservation (OTC), ovarian reserve, pregnancy, oncofertility

## Abstract

Immune checkpoint inhibitors (ICIs) have reshaped melanoma care, yielding durable remissions even in high-risk stages. As survival improves, fertility becomes a key survivorship concern for young women, yet the reproductive safety of ICIs remains insufficiently characterised. We performed a SWOT (Strengths, Weaknesses, Opportunities, Threats) analysis synthesizing preclinical and clinical evidence to evaluate the rationale for fertility preservation (FP) prior to checkpoint inhibitor therapy. Preclinical models of PD-1/CTLA-4 blockade demonstrate ovarian immune activation, cytokine release (e.g., TNF-α), and follicular loss. Conversely, human data are limited to correlative analyses suggesting potential declines in ovarian reserve markers. In conclusion, while prospective studies are required to definitively quantify risk, proactive fertility preservation counselling should be routinely offered prior to treatment initiation to safeguard reproductive autonomy without compromising oncologic safety.

## Simple Summary

Whether pre-treatment fertility counselling should be routinely offered to reproductive-age women with melanoma who are candidates for immune checkpoint inhibitors remains uncertain. These agents mandate contraception during therapy and a post-treatment washout, while evidence on ovarian reserve and fertility outcomes is still limited. This SWOT (Strengths, Weaknesses, Opportunities, and Threats) analysis evaluates the rationale for fertility preservation prior to checkpoint inhibitor therapy. It integrates current evidence with the practical timing constraints of standard perioperative pathways. It weighs benefits and risks, clarifies areas of uncertainty—including pregnancy and breastfeeding near exposure—and identifies practical levers for services (early referral, clear documentation, and follow-up of menstrual and hormonal parameters). The goal is to support evidence-aware counselling and time-sensitive decision-making, and to inform prospective data collection and quality improvement in melanoma care.

## 1. Introduction

Cutaneous melanoma is a major malignancy among adolescents and young adults (AYAs, 15–39 years), with the highest incidence observed in very-high and high-income regions; in Europe it ranks among the most common AYA cancers [1,2,3]. Over the past decade, care has been transformed by immune checkpoint inhibitors (ICIs). Anti-PD-1 and anti-CTLA-4 antibodies are now standard components of therapy in advanced disease and in surgically resected high-risk settings. In resected stage IIB–IIC melanoma, adjuvant pembrolizumab improves recurrence-free and distant-metastasis–free survival (KEYNOTE-716). In stage III–IV disease, adjuvant nivolumab has replaced ipilimumab due to superior efficacy and tolerability (CheckMate-238). Finally, perioperative PD-1 therapy provides additional benefit in resectable stage III/IV disease (SWOG S1801) [4,5,6,7]. As more young women achieve durable remission and consider pregnancy, reproductive safety under ICIs has become a priority. Endocrine immune-related adverse events—especially thyroid dysfunction and hypophysitis—are well described, but the long-term effect of ICIs on ovarian reserve and fertility potential remains unknown [8,9,10,11]. Direct human evidence is limited: in a correlative analysis from the US phase III ECOG-ACRIN E1609 adjuvant trial of ipilimumab, young women showed post-treatment declines in anti-Müllerian hormone and other ovarian-reserve markers [12]. However, the EMA has not approved this indication [13]. Consequently, adjuvant ipilimumab is not part of standard adjuvant melanoma therapy in Europe. Instead, PD-1 inhibitors (nivolumab, pembrolizumab) have become the preferred adjuvant options based on phase III trial results and current EMA-approved indications [5,14,15]. In parallel, preclinical studies suggest that PD-L1/CTLA-4 blockade can trigger intra-ovarian immune activation with cytokine release, follicular loss and oocyte impairment [16,17,18]. These gaps motivate a SWOT analysis. This framework was specifically selected to synthesise heterogeneous evidence streams—ranging from preclinical mechanisms to clinical logistics—into an actionable decision-making tool. By integrating biological rationale with practical opportunities and threats, this analysis aims to support evidence-aware counselling and survivorship planning in a complex clinical setting.

## 2. Materials and Methods

To inform this SWOT analysis, a focused evidence synthesis was performed by querying PubMed/MEDLINE, major oncology and reproductive medicine guideline repositories, and EMA/FDA product information. No publication date restrictions were applied, and the search covered the literature up to 12 October 2025. Search algorithms incorporated combinations of the following keywords: “melanoma”, “immune checkpoint inhibitors”, “pembrolizumab”, “nivolumab”, “ipilimumab”, “endocrine adverse events”, “hypophysitis”, “thyroiditis”, “ovarian reserve”, “AMH”, “fertility preservation”, “pregnancy”, and “oocyte/ovarian tissue cryopreservation”.

Selection prioritised clinical and preclinical studies, guidelines, and meta-analyses. Specifically, the analysis included the available clinical datasets evaluating ovarian reserve under ICIs (e.g., the ECOG-ACRIN E1609 analysis), endocrine irAE meta-analyses, and preclinical studies on the ovarian effects of checkpoint blockade. Sources were critically appraised for their methodological quality and clinical applicability. For clinical evidence, we preferentially included phase II–III trials, prospective cohorts, and systematic reviews; smaller case series and narrative reviews were used to contextualise, but not to drive, the main conclusions. National and international guidelines from major societies (e.g., ESMO, ASCO, ESHRE/ESE) and EMA/FDA labels were considered high-quality evidence sources. Articles not available in English or lacking specific relevance to the topic were excluded. Screening and synthesis were performed by the authors; no quantitative pooling was attempted. A summary of the included evidence sources is provided in Table 1. As this is a focused narrative review, reporting was structured to address key domains highlighted in tools for narrative reviews, including the SANRA (Scale for the Assessment of Narrative Review Articles) items on literature search description, adequacy of referencing, and transparent presentation of the level of evidence.

## 3. Biological Rationale and Clinical Evidence

ICIs may affect female reproduction via two pathways: immune-mediated ovarian injury and immune-related endocrine dysfunction. In murine models, anti-CTLA-4 and anti-PD-1/PD-L1 therapies drive CD3^+^ T-cell infiltration, increase intra-ovarian cytokines (e.g., TNF-α), and deplete the primordial follicle pool. These changes impair oocyte maturation and ovulation, providing a plausible link between checkpoint blockade and ovarian damage [16,17]. Because PD-1 signalling underpins peripheral tolerance, its blockade could permit autoreactivity in predisposed individuals, although specific ovarian targets remain undefined [48]. In addition to potential direct ovarian injury, immune-related endocrine adverse events (irAEs) represent a significant clinical burden. Hypophysitis occurs more often with CTLA-4–containing or combination regimens than with PD-1/PD-L1 monotherapy and typically results in secondary hypogonadism and other pituitary hormone deficiencies which are frequently long-lasting and irreversible, requiring lifelong hormone replacement in most patients [8,40]. Thyroiditis and hypothyroidism (occasionally hyperthyroidism) are among the most frequent endocrine irAEs with ICIs; meta-analyses report hypothyroidism ≈ 3.8% with CTLA-4 monotherapy (ipilimumab), ≈7% with PD-1 monotherapy (6.5% with nivolumab; 7.9% with pembrolizumab), and 13.2% with combined regimens; the pooled overall incidence across regimens is 6.6% [8,9]. While generally manageable, these disturbances can complicate attempts to conceive. Human evidence regarding ovarian reserve is sparse. In the ECOG-ACRIN E1609 trial, women receiving ipilimumab showed post-treatment declines in AMH, estradiol and luteinising hormone, with stable FSH and prolactin; however, the small sample size and the ipilimumab-specific setting limit generalisability, and data for PD-1/PD-L1 inhibitors are lacking [12]. A recent narrative review underscores the paucity of robust clinical evidence, highlights these unmet needs in melanoma-specific fertility preservation, and calls for well-designed prospective studies [19]. Taken together, current data suggest possible subclinical changes in ovarian markers in small cohorts, but the direction, magnitude and impact on fertility remain undefined. This uncertainty warrants precautionary counselling, consideration of fertility preservation, and ovarian reserve monitoring during follow-up [11,12,19]. Prospective, AYA-focused cohorts embedded within adjuvant or perioperative ICI programmes could define trajectories of reproductive function by serially assessing AMH, AFC, menstrual patterns and subsequent pregnancy outcomes under PD-1/PD-L1 or CTLA-4 blockade [4,5,6,12]. Pre-specified sampling windows aligned with treatment timelines and harmonised case-report elements would reduce measurement variability and facilitate cross-centre comparability, while documenting the real-world feasibility of fertility preservation in surgical and perioperative settings [52,53,56]. In this context, the MELAFERT trial (NCT07092670)—a prospective multicentre observational study promoted by the Intergruppo Melanoma Italiano and currently ongoing in Italy—specifically investigates the impact of adjuvant MAP kinase pathway inhibitors and ICIs on fertility in patients with resected high-risk melanoma and is expected to generate prospective data to support patient counselling and fertility-preservation implementation [45].

## 4. SWOT Analysis

**Figure 1:**
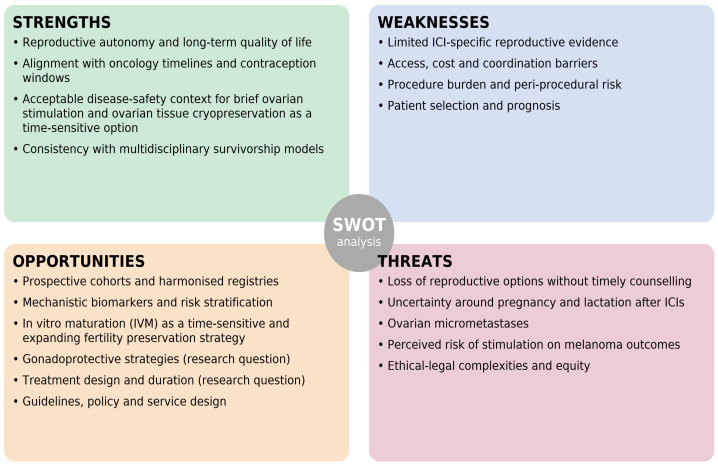
SWOT analysis summarising key factors related to fertility preservation in young women with melanoma receiving immune checkpoint inhibitors.

A synthetic overview of the main clinical implications for each quadrant is presented in Table 2, while a graphical summary of the SWOT analysis is provided in Figure 1.

### 4.1. Strengths

#### 4.1.1. Reproductive Autonomy and Long-Term Quality of Life

Early fertility-preservation (FP) counselling preserves future reproductive options and is consistently associated with lower decisional regret and better long-term well-being among young cancer survivors [23,52]. In AYA melanoma, where durable remissions are increasingly common, FP directly addresses a core survivorship priority: the possibility of biological parenthood after treatment [24]. Integrating FP into the initial oncology pathway allows patients to make informed choices before starting ICIs, when timelines still permit action [31]. Consensus guidance from oncology and reproductive societies positions timely FP referral as standard good practice, reinforcing that counselling is not an “optional add-on” but part of comprehensive cancer care [56]. Structured FP counselling strengthens reproductive autonomy by providing transparent, evidence-based estimates of treatment-related reproductive risk and realistic probabilities of FP success [23,52]. It improves knowledge, preparedness, and decisional quality and is consistently associated with lower decisional conflict and regret as well as better patient-reported outcomes during survivorship [24,31]. Clear expectation-setting about future family-building—including the potential need for ART—and explicit communication of the residual uncertainty surrounding ICI-related gonadotoxicity mitigate anxiety and support coherent life planning after melanoma treatment [31]. Systematic documentation of counselling content and preferences facilitates continuity across specialties and over time, and when offered systematically to all eligible patients using standardized information resources, reduces unwarranted variability and promotes equitable, patient-centred care [56].

#### 4.1.2. Alignment with Oncology Timelines and Contraception Windows

Pregnancy is contraindicated during immune checkpoint blockade and requires a post-treatment washout—approximately 4 months for pembrolizumab [15], 5 months for nivolumab [14], and 3 months for ipilimumab [13]. Planning FP before starting ICIs creates a realistic path to conception once disease control is achieved and the contraception window has elapsed [52]. Modern random-start stimulation enables immediate initiation regardless of cycle phase, with retrieval in approximately 10–14 days [21,53]; this typically fits the post-operative interval before adjuvant therapy. Importantly, major adjuvant and perioperative trials (e.g., KEYNOTE-716, CheckMate-238, SWOG S1801) allow several weeks from surgery to treatment start—time that can be used for FP without compromising oncologic intent [4,5,6,52,53].

Coordinating FP procedures within this perioperative timeline requires close communication between surgical, medical oncology and reproductive teams. Standard operating procedures specifying the interval from surgery to first adjuvant infusion, contraception counselling and FP referral timing can prevent missed opportunities. Evidence from trial protocols confirms that up to ~12 weeks are routinely allowed from surgery to treatment start—time that can be used for FP without compromising oncologic intent [4,5,6,21,53]. Integrating these data into institutional pathways reassures clinicians that FP can be safely completed without delaying systemic therapy. Moreover, clear documentation of the planned washout and contraception period facilitates coordination of survivorship counselling and subsequent pregnancy planning [23,24,52]. This pragmatic alignment of oncologic and reproductive timelines exemplifies multidisciplinary care and enhances both treatment adherence and long-term quality-of-life outcomes.

Neoadjuvant and response-directed strategies are also rapidly emerging and may become prevalent over purely adjuvant approaches. Phase II programmes such as OpACIN-neo and its PRADO extension have shown that two cycles of neoadjuvant ipilimumab plus nivolumab can induce high pathologic response rates and allow response-directed surgery and adjuvant therapy in stage III melanoma [41,59]. More recently, the phase III NADINA trial demonstrated that neoadjuvant ipilimumab plus nivolumab followed by surgery and response-adapted adjuvant therapy resulted in superior event-free survival compared with the standard sequence of surgery followed by adjuvant nivolumab [47]. In this evolving scenario, systemic therapy is brought earlier in the treatment pathway, further compressing the window for FP between diagnosis, surgery and systemic treatment. Early identification of FP candidates at the time of diagnosis, and streamlined referral to oncofertility services, therefore becomes even more critical in neoadjuvant and perioperative ICI programmes.

#### 4.1.3. Acceptable Disease-Safety Context for Brief Ovarian Stimulation and Ovarian Tissue Cryopreservation as a Time-Sensitive Option

Cutaneous melanoma is not considered hormone-driven, and available data do not show worse melanoma prognosis with subsequent pregnancy [25,26] nor an increased melanoma incidence after ovarian stimulation for IVF [27]. The low and heterogeneous expression of estrogen receptors in melanoma further reduces the biological plausibility that transient estradiol rises during controlled ovarian stimulation would influence disease control [49]. Taken together with contemporary oncology/reproductive guidance, these observations support the acceptability of a short, controlled estrogen exposure for fertility preservation (FP) in this population [52].

Although melanoma is not estrogen-driven, many oncofertility programs use letrozole-supplemented stimulation to minimise estradiol exposure during FP; this precautionary approach is guideline-concordant and does not compromise oocyte yield, yet it is primarily recommended for estrogen-sensitive malignancies, and antagonist random-start stimulation without letrozole remains acceptable in melanoma with individualisation of care [20,21,52,53]. When time permits, the primary FP options are controlled ovarian stimulation, ovarian tissue cryopreservation (OTC), or both within the same perioperative window according to patient preference [22,52,53,56]. When controlled stimulation is infeasible or decision-to-treatment intervals are too short, OTC is a guideline-endorsed alternative that can be arranged immediately and may preserve both endocrine and reproductive potential [53,56]. When time permits, a combined approach can be planned within the same perioperative window, coupling OTC and brief random-start stimulation, to maximise the number of cryopreserved gametes without delaying immunotherapy initiation [22,52,56].

In addition, in vitro maturation of oocytes (IVM) should be considered as an adjunct (an emerging, non–non-standard-of-care option) when immature oocytes are obtained or when only a short stimulation is feasible. Specifically, immature oocytes from the excised cortex at the time of OTC can undergo OTO-IVM, while those retrieved during a brief controlled stimulation can undergo rescue-IVM. This strategy increases the number of mature (MII) oocytes without prolonging treatment [21,53,56]. Notably, IVM (including OTO-IVM) has lower and more variable success rates than conventional IVF, and current oncology data are largely observational; therefore, its use should be individualised and depend on local expertise [56].

#### 4.1.4. Consistency with Multidisciplinary Survivorship Models

Integrating fertility preservation (FP) into routine oncology visits aligns with major society recommendations to prioritize reproductive health as a standard component of comprehensive cancer care [52,53,56]. Structured, multidisciplinary models—connecting oncologists, reproductive specialists, and psychosocial staff—ensure consistent delivery of contraception and fertility guidance while reducing missed opportunities. Simple operational tools, such as electronic referral prompts and standardized survivorship templates, facilitate documentation and foster continuity of care beyond the active treatment phase [31,56]. This integration exemplifies patient-centred oncology, bridging medical efficacy with long-term psychosocial recovery [31,52].

### 4.2. Weaknesses

#### 4.2.1. Limited ICI-Specific Reproductive Evidence

Human data directly assessing ovarian reserve and fertility outcomes under immune checkpoint inhibitors (ICIs) remain limited. The only prospective signal comes from the ECOG-ACRIN E1609 adjuvant programme with ipilimumab, where young women exhibited post-treatment declines in AMH and selected hormones; however, the small sample, agent specificity, and lack of validated fertility endpoints (e.g., time-to-pregnancy, live birth) preclude causal inference [12]. For PD-1/PD-L1 monotherapy, there is no robust prospective evidence quantifying trajectories of AMH/AFC or subsequent reproductive outcomes; current knowledge largely rests on mechanistic plausibility and expert opinion summarised in recent reviews, including a melanoma-specific oncofertility overview [11,28,29,46].

Translational gaps further limit interpretability. Preclinical models demonstrate intra-ovarian immune activation, pro-inflammatory cytokine shifts (e.g., TNF-α), apoptosis-related follicular loss, and oocyte impairment under PD-1/PD-L1 or CTLA-4 blockade. However, species, dosing, and immune-context differences complicate extrapolation to humans [16,17,18]. In clinical cohorts, endocrine immune-related adverse events (e.g., thyroid dysfunction, hypophysitis) are relatively frequent and may confound ovarian-reserve markers or menstrual patterns without directly reflecting gonadal injury, making attribution challenging [8,9,10]. Additional sources of bias include heterogeneity in baseline ovarian reserve, age distribution, prior surgeries, assay variability, and sparse longitudinal sampling.

Overall, the evidence base is suggestive but insufficient to define the magnitude, direction, or persistence of ICI-related reproductive risk in melanoma. This justifies precautionary counselling and structured monitoring while prioritising prospective, melanoma-specific cohorts with pre-specified reproductive endpoints and harmonised sampling schedules to close current knowledge gaps [11,12,28,29].

#### 4.2.2. Access, Cost and Coordination Barriers

FP costs, coverage and eligibility criteria vary substantially across health systems, resulting in unequal access—particularly for adolescents and young adults and for patients managed outside tertiary centres [30,54]. Even when public or private coverage is available, time-sensitive coordination across surgical, medical oncology and reproductive teams is challenging, especially in settings that lack dedicated oncofertility services or formal pathways [31,56]. Inconsistent referral practices and limited local capacity have been associated with delayed or missed FP offers, reducing timely uptake before cancer treatment [30,56].

Current guidance emphasises early FP counselling at diagnosis, timely referral to reproductive services, and the integration of reproductive health within multidisciplinary cancer care and survivorship planning [31,52,56]. These documents also highlight the need to record reproductive counselling—including contraception during therapy, post-treatment washout, and future fertility considerations—as part of comprehensive care [31,52,56]. Where available, regional or multicentre collaborations can facilitate access and help reduce geographic inequities by connecting oncology units with experienced reproductive programs [31,54].

These barriers are often exacerbated in neoadjuvant settings, where systemic therapy is initiated earlier in the treatment pathway and the interval between diagnosis, surgery and the first infusion is intrinsically short [4,5,6,41,47,59]. In this setting, oncofertility referral ideally needs to occur at or shortly after diagnosis, with fast-track access to reproductive consultations and predefined time slots for FP procedures, to ensure that eligible women can complete FP without delaying neoadjuvant or perioperative immunotherapy. Incorporating explicit references to neoadjuvant regimens and compressed timelines into institutional pathways can help align access, cost coverage and coordination efforts with real-world melanoma treatment schedules [30,31,52,54,56].

#### 4.2.3. Procedure Burden and Peri-Procedural Risk

Fertility preservation with controlled ovarian stimulation involves daily injections, repeated ultrasound/biochemical monitoring, and a transvaginal oocyte retrieval under procedural sedation; while generally well tolerated, this sequence adds appointments and procedural demands within a time-sensitive oncologic pathway [31,52,53]. Serious complications are uncommon, but ovarian hyperstimulation syndrome (OHSS) remains the principal procedure-related concern in fertility preservation; prevention and risk-mitigation strategies are recommended in dedicated guidance and should be incorporated into protocols [55]. In the setting of stimulation undertaken before potentially gonadotoxic therapy, the priority is to minimise stimulation-related OHSS while preserving oocyte yield. Recommended measures include the use of antagonist protocols with a GnRH-agonist trigger for final oocyte maturation, individualised gonadotropin dosing according to ovarian reserve, and—when indicated—dopamine-agonist prophylaxis (e.g., cabergoline), alongside close monitoring with readiness to modify or cancel the cycle in case of excessive response [21,53,55]. When timing conflicts arise or when ovarian response is inadequate or excessive, cycle modification or cancellation may be required, underscoring the need for schedules that accommodate rapid initiation and close monitoring typical of oncofertility settings [21].

Psychological and logistical load at diagnosis can influence acceptance and adherence; therefore, guidance documents emphasise anticipatory information on the expected course of stimulation and peri-procedural management as components of comprehensive cancer care [31,52,53,56].

#### 4.2.4. Patient Selection and Prognosis

Fertility preservation (FP) should be considered in relation to disease stage, anticipated time-to-treatment, and realistic prospects for future use of cryopreserved gametes, within a shared decision-making process [31,56]. In patients with rapidly progressive disease, limited life expectancy, or when treatment timelines preclude timely initiation of stimulation, FP may not be feasible or appropriate [31]. Guidance documents emphasise that counselling should integrate prognosis and therapeutic schedules and be delivered within multidisciplinary pathways [31,56].

In melanoma specifically, candidacy for FP differs across clinical settings. In resected high-risk disease eligible for adjuvant immunotherapy, defined perioperative windows before treatment initiation are commonly available according to trial protocols (up to ~12 weeks), which may allow one FP cycle when planned promptly [4,5,6,52]. By contrast, in advanced disease requiring urgent systemic therapy or in the presence of high symptomatic burden, the feasibility of FP is reduced by clinical urgency and narrower timelines [31]. Accordingly, decisions should account for expected treatment windows, oncologic priorities, and the probability that stored oocytes/embryos will be used in the future, aligning reproductive planning with survivorship expectations [31,56].

### 4.3. Opportunities

#### 4.3.1. Prospective Cohorts and Harmonised Registries

Prospective, AYA-focused cohorts embedded within adjuvant or perioperative ICI programmes can define trajectories of reproductive function by serially assessing AMH, AFC, menstrual patterns and subsequent pregnancy outcomes under PD-1/PD-L1 or CTLA-4 blockade [4,5,6,12]. Pre-specified sampling windows aligned with treatment timelines and harmonised case-report elements would reduce measurement variability and facilitate cross-centre comparability, while documenting the real-world feasibility of FP in surgical and perioperative settings [52,53,56].

Harmonised registries using common data models could enable multicentre pooling and faster learning, linking reproductive endpoints with oncology variables (stage, regimen, endocrine irAEs) to improve inference and distinguish direct ovarian effects from endocrine confounding [24,52,53,56]. Such infrastructures would also support consistent capture of counselling and referral timing, FP uptake, and post-treatment reproductive outcomes, providing the basis for robust, melanoma-specific evidence to guide counselling and service planning [52,53,56].

#### 4.3.2. Mechanistic Biomarkers and Risk Stratification

Preclinical studies under PD-1/PD-L1 or CTLA-4 blockade demonstrate intra-ovarian immune activation, pro-inflammatory cytokine shifts, and follicular loss, thereby establishing a biological basis for ICI-associated ovarian alterations [16,17,18]. Translating these mechanistic signals into clinical practice requires prospective biomarker frameworks. These frameworks should combine reproductive markers (AMH, menstrual patterns) with immune readouts, such as cytokine profiling (e.g., TNF-α), peripheral immune phenotyping, and exploratory anti-ovarian autoantibodies. This approach allows researchers to characterise trajectories and distinguish direct ovarian effects from endocrine confounding [12,16,17,18].

Embedding pre-specified sampling windows aligned with treatment timelines (baseline and on-treatment timepoints, plus post-therapy follow-up) would reduce variability and allow correlation of biomarker dynamics with clinical variables such as endocrine irAEs, stage, and treatment regimen [4,5,6,12]. Within this structure, AMH/AFC trends can serve as pragmatic surveillance anchors, while immune and serologic panels provide mechanistic context and help identify subgroups warranting closer monitoring or earlier FP referral [12,16,17,18].

Harmonised case-report elements across centres—covering assay methodology, sampling timepoints, and definitions of menstrual and endocrine events—are essential to enable data pooling and comparability, and to clarify whether observed hormonal shifts primarily reflect ovarian injury, central endocrine irAEs, or both [11,12,16,17,18,53,56]. These datasets would provide a melanoma-specific evidence base to inform counselling, refine risk stratification, and calibrate surveillance intensity during and after ICIs [12,16,17,18,53,56].

#### 4.3.3. In Vitro Maturation (IVM) as a Time-Sensitive and Expanding Fertility Preservation Strategy

As outlined above, IVM–including OTO-IVM and rescue-IVM after stimulation–is variably available and its outcomes remain more heterogeneous than conventional IVF in the oncology setting, with an evidence base largely derived from small series [56]. Recent guidance recognises IVM as a clinically applicable, emerging FP option in selected patients, while noting limited availability and heterogeneous outcomes compared with conventional IVF [53,56]. In time-sensitive oncology pathways, any evaluation or use of IVM should be organised so as not to delay systemic therapy, with planning aligned to treatment schedules [53,56]. This profile identifies IVM as a priority for research and service improvement rather than routine use: prospective data collections with harmonised reporting of key laboratory and clinical endpoints are needed to benchmark performance across centres [21,53].

Harmonised frameworks should specify core data elements, including population descriptors (age, ovarian reserve, cancer stage), exposure definitions (IVM modality and timing), and laboratory conditions (culture system, maturation interval). They must also define standard endpoints: GV-to-MII conversion, MII yield per patient, fertilisation method and rate, blastocyst formation, cryosurvival after warming, and subsequent clinical outcomes. Finally, frameworks should include safety metrics and follow-up windows compatible with oncology timelines [21,53,56]. Such standardisation would reduce case-mix and methodological heterogeneity and enable meaningful cross-centre comparisons, including the distinction between OTO-IVM and rescue-IVM cohorts.

Where OTC is undertaken, coordinated capture of OTO-IVM procedures within institutional or multicentre registries can clarify feasibility and incremental value without delaying immunotherapy initiation, linking reproductive endpoints to perioperative and oncologic variables [22,53,56]. In parallel, embedding IVM activity within these registries provides a practical platform for continuous quality improvement and transparent counselling, helping to define the clinical contexts in which IVM adds value within modern oncofertility pathways [21,22,53,56].

#### 4.3.4. Gonadoprotective Strategies (Research Question)

In melanoma, selective biologics such as infliximab or vedolizumab are sometimes required to treat severe immune-related colitis that does not respond to corticosteroids. Observational series have not shown a consistent negative impact on tumour control when these agents are used for clinically indicated irAEs, providing a relevant clinical context for exploratory reproductive research [32,33,34]. Preclinical data show that TNF-α can directly trigger oocyte/follicle apoptosis in the neonatal rat ovary, and that TNF-α–driven processes can reduce ovulation and induce granulosa cell death in vivo, supporting biological plausibility that cytokine-driven inflammation may be relevant to ovarian function [50,51]. In addition, age-related ovarian follicle depletion in mice has been associated with intra-ovarian increases in pro-inflammatory cytokines, including TNF-α [58]. Within this strictly indication-driven setting, prospective observational cohorts could compare trajectories of ovarian-reserve markers and menstrual function in patients who receive biologics for colitis versus corticosteroids alone, while tracking oncologic endpoints to exclude trade-offs in efficacy [32,33,34,50,51,58].

#### 4.3.5. Treatment Design and Duration (Research Question)

Current adjuvant PD-1 strategies in melanoma—such as pembrolizumab or nivolumab administered for approximately 12 months—are fixed in duration, regardless of early pathological or molecular response [4,5]. This schedule extends the interval before conception can be safely attempted, as reproductive planning must include both the treatment course and the required post-therapy washout interval [13,14,15]. In young women wishing to preserve fertility potential, prolonged exposure and delayed family planning may represent a major survivorship concern.

Long-term clinical follow-up in metastatic melanoma indicates that antitumor effects can persist after PD-1 discontinuation, raising the hypothesis that shorter adjuvant courses might maintain efficacy while reducing cumulative exposure [42,43]. Response-adapted or risk-stratified designs—guided by pathological complete response, minimal residual disease negativity, or early immune signatures—could therefore identify subgroups for whom de-escalation is oncologically safe and reproductively advantageous [41]. Any such approach must be evaluated prospectively, within randomised trials with robust recurrence-free and overall survival endpoints and accompanied by predefined reproductive metrics and safety monitoring. Current evidence from metastatic settings cautions that abbreviated exposure may affect durability of response; therefore, treatment de-escalation should remain a research question and not a routine practice outside clinical trials [35,42,43].

#### 4.3.6. Guidelines, Policy and Service Design

Existing frameworks (ASCO/ESMO/ESHRE) already endorse early FP referral. Incorporating ICI-specific language—washout windows, endocrine irAE monitoring relevant to reproduction, and documentation standards—could improve consistency. Policies enabling equitable reimbursement and automatic referral triggers (e.g., at ICI ordering) may reduce access gaps. Prospective service audits that include time-to-referral, uptake, complications, live-birth and time-to-pregnancy can drive quality improvement [31,52,56].

A concise label-based summary is provided (Table 3).

### 4.4. Threats

#### 4.4.1. Loss of Reproductive Options Without Timely Counselling

Underestimation of potential gonadal toxicity—or age-related decline during prolonged treatment—may result in the irreversible loss of reproductive potential. In young survivors, omitted or delayed FP counselling is linked to persistent decisional regret and lower quality of life, a preventable survivorship burden. Practical bottlenecks (postoperative recovery, pathology turnaround, insurance approvals, MDT scheduling) compress the pre-adjuvant window and can make it infeasible to organise stimulation and cryopreservation without delaying systemic therapy. Washout requirements after adjuvant ICIs (drug-specific) further extend time to conception, compounding age-related decline and partner-/gamete-availability constraints. Standardised, early referral pathways and auditable documentation reduce the likelihood of missing the short interval between surgery and treatment start [13,14,15,23,24,52].

#### 4.4.2. Uncertainty Around Pregnancy and Lactation After ICIs

Product information mandates strict contraception during therapy and a drug-specific post-treatment washout; breastfeeding is generally discouraged during treatment and for a defined interval thereafter [13,14,15]. Available pharmacovigilance and case series do not show a uniform pattern of specific maternofetal toxicity versus other anticancer drugs; however, signals such as higher rates of preterm birth with PD-1 plus CTLA-4 combinations sustain a cautious stance. Risk is heterogeneous and time-dependent (pre-conception exposure vs. first vs. late gestation), with potential for Fc-mediated transplacental transfer later in pregnancy and limited neonatal safety data. In practice, heterogeneous outcomes in reported cases—many in melanoma—reinforce case-by-case assessment, explicit documentation of uncertainties, and liaison with maternal-fetal medicine; lactation decisions should mirror label language within shared decision-making [13,14,15,38,39,44,57].

#### 4.4.3. Ovarian Micrometastases

Clinicopathologic series describe ovarian involvement by melanoma—sometimes years after the primary tumour—with immunohistochemistry (S-100, HMB-45, MART-1/Melan-A, ±tyrosinase/MiTF) aiding diagnosis. For FP strategies entailing tissue reimplantation (e.g., ovarian cortex), centre-level SOPs should include oncologic vetting, rigorous pathological review and validated handling to minimise reseeding risk. Where residual oncologic risk cannot be acceptably mitigated, preference should shift toward FP modalities that do not require reimplantation. The absolute risk is low but non-zero and must be made explicit during counselling, in line with reproductive-oncology guidance [36,37,52].

#### 4.4.4. Perceived Risk of Stimulation on Melanoma Outcomes

Large population-based cohorts do not show increased melanoma incidence after ART/ovarian stimulation, which is reassuring from a causation standpoint. Nevertheless, recurrence-specific data immediately after ICIs are limited; perceived oncologic risk can deter referrals, prolong indecision or delay time-sensitive FP steps. Mitigation relies on multidisciplinary alignment with oncology, explicit communication of what is known vs. unknown, selection of stimulation schedules that avoid clinically unacceptable postponements of adjuvant therapy, and clear documentation of the rationale when FP is deferred [27].

#### 4.4.5. Ethical-Legal Complexities and Equity

Offering FP in the setting of very poor prognosis may be perceived as overtreatment or as fostering unrealistic expectations; conversely, failing to offer FP to eligible patients can lead to lasting regret and complaints. Transparent documentation, stage-specific recommendations and structured shared decision-making help navigate these tensions. From an equity perspective, coverage gaps, regional variability in referral practices, and logistical barriers (travel, time-off, childcare) disproportionately affect vulnerable patients. Implementing standardised referral triggers and auditable counselling notes can reduce this variability in access and outcomes [23,24,31,52,56].

## 5. Conclusions

Emerging evidence—largely preclinical and observational—suggests that ICIs may exert subclinical effects on ovarian function, while definitive clinical risk quantification remains pending. In parallel, pregnancy and lactation during or shortly after ICIs warrant caution, and ovarian tissue–based strategies require oncologic vetting given the low but non-zero risk of ovarian involvement in melanoma. Conversely, current population-based data are reassuring regarding melanoma incidence after ovarian stimulation, and the principal near-term threat is the loss of reproductive options when counselling is delayed.

Against this backdrop, fertility preservation (FP) should be discussed proactively with young women before immunotherapy, using standardised documentation, automatic referral pathways where available, and coordination that avoids delaying adjuvant therapy. When reimplantation-free options are feasible, they should be prioritised in higher-risk scenarios; survivorship care should include ovarian reserve and menstrual function monitoring and clear guidance for pregnancy planning and breastfeeding.

A focused research agenda is now required: multicentre prospective cohorts and registries with predefined reproductive endpoints; response-adapted adjuvant strategies tested within trials that include fertility and obstetric outcomes; and mechanistic studies clarifying inflammatory pathways and endocrine irAEs in ovarian biology. Until higher-level evidence is available, patient-centred counselling and individualised care pathways offer the best alignment of oncologic success with long-term reproductive goals.

## Figures and Tables

**Table 1:** Evidence sources included in the focused narrative synthesis informing the SWOT analysis, grouped by type of evidence.

Evidence Type	Scope and Inclusion Criteria	Number of Sources (N)	References
Clinical human studies	Randomized trials and real-world cohorts of immune checkpoint inhibitors in melanoma; epidemiology and survival in adolescents and young adults; clinical and pathological series on ovarian metastases; endocrine irAEs, ovarian reserve and fertility-related outcomes under ICIs; fertility preservation techniques and utilisation (across cancers and in melanoma); pregnancy and melanoma; pregnancy and lactation after ICIs; ICI-induced colitis and selective immunosuppression; oncofertility implementation studies; ClinicalTrials.gov record of the ongoing MELAFERT trial	41	[1,2,3,4,5,6,7,8,9,10,12,18,19,20,21,22,23,24,25,26,27,28,29,30,31,32,33,34,35,36,37,38,39,40,41,42,43,44,45,46,47]
Pre-clinical/animal and mechanistic studies	Murine and rat models exploring PD-1/PD-L1 blockade, immune-related cardiomyopathy, tumour necrosis factor-α–mediated oocyte/follicle apoptosis, and inflammation-associated follicle depletion in the ovary, used to infer potential mechanisms of gonadotoxicity under immune checkpoint inhibition.	6	[16,17,48,49,50,51]
Guidelines, regulatory documents and practice frameworks	EMA product information for nivolumab, pembrolizumab and ipilimumab; ASCO, ESMO and ESHRE clinical practice guidelines on fertility preservation and on cancer during pregnancy; ASRM guideline on prevention and management of ovarian hyperstimulation syndrome; endocrine society guideline on immune-related endocrine AEs; LactMed monograph on pembrolizumab;	11	[11,13,14,15,52,53,54,55,56,57,58]

**Table 2:** Synthetic overview of the SWOT analysis findings and clinical implications.

SWOT Section	Main Clinical Synthesis and Implications
Strengths	Autonomy and Feasibility: Early counselling preserves reproductive autonomy. Random-start ovarian stimulation is feasible within the standard post-operative windows of adjuvant therapy (up to ~12 weeks) without delaying treatment. The procedure is considered oncologically safe as melanoma is not hormone-driven.
Weaknesses	Specific Evidence Gaps and Barriers: Clinical data on ovarian reserve are limited to ipilimumab (ECOG-ACRIN E1609), which is not standard adjuvant therapy in Europe, while data on PD-1 inhibitors are lacking. A major barrier is the compressed timeline in emerging neoadjuvant settings, where systemic therapy is initiated at diagnosis, leaving a narrower window for fertility preservation.
Opportunities	Prospective Research and Innovation: Ongoing prospective studies (e.g., the MELAFERT trial) are essential to generate real-world evidence on fertility outcomes. Harmonised registries and neoadjuvant platforms offer opportunities to define risk stratification. IVM (In Vitro Maturation) represents a potential time-sensitive strategy for patients with urgent treatment needs.
Threats	Missed Care and Uncertainty: The principal threat is the loss of reproductive potential due to delayed referral (“missed opportunities”). Management is complicated by the lack of safety data on pregnancy and lactation during/after ICIs, and by the theoretical risk of ovarian micrometastases in tissue reimplantation strategies.

**Table 3:** Label-based contraception, washout and lactation guidance for ICIs relevant to melanoma (Product labels: EMA SmPC/FDA PI).

Drug	Contraception During Therapy	Post-Treatment Washout (Minimum)	Breastfeeding Recommendation
Pembrolizumab	Required	≥4 months after last dose	Avoid during treatment and for ≥4 months after last dose
Nivolumab	Required	≥5 months after last dose	Avoid during treatment and for ≥5 months after last dose
Ipilimumab	Required	≥3 months after last dose	Avoid during treatment and for ≥3 months after last dose

## Data Availability

No new data were created or analyzed in this study. Data sharing is not applicable to this article.

## Abbreviations

The following abbreviations are used in this manuscript:

**Abbreviation**	**Definition**
AFC	Antral follicle count
AMH	Anti-Müllerian hormone
ART	Assisted reproductive technology
ASCO	American Society of Clinical Oncology
AYA	Adolescents and young adults
COS	Controlled ovarian stimulation
CTLA-4	Cytotoxic T-lymphocyte–associated protein 4
ECOG-ACRIN	Eastern Cooperative Oncology Group–American College of Radiology Imaging Network
EMA	European Medicines Agency
ESE	European Society of Endocrinology
ESHRE	European Society of Human Reproduction and Embryology
ESMO	European Society for Medical Oncology
FDA	Food and Drug Administration
FET	Frozen embryo transfer
FP	Fertility preservation
GnRH	Gonadotrophin-releasing hormone
GV	Germinal vesicle
ICI	Immune checkpoint inhibitor
irAE	Immune-related adverse event
IVF	In vitro fertilisation
IVM	In vitro maturation
MDT	Multidisciplinary team
MII	Metaphase II (oocyte stage)
OHSS	Ovarian hyperstimulation syndrome
OTC	Ovarian tissue cryopreservation
OTO-IVM	Oocyte in vitro maturation from ovarian tissue
PD-1	Programmed cell death protein 1
PD-L1	Programmed death-ligand 1
PI	Prescribing information
SmPC	Summary of Product Characteristics
SWOT	Strengths, Weaknesses, Opportunities, and Threats
TNF-α	Tumour necrosis factor-alpha
